# Role of Anharmonic Interactions for Vibration Density of States in α-Cristobalite

**DOI:** 10.3390/ma14030617

**Published:** 2021-01-29

**Authors:** Yongda Huang, Jian Zhou, Guanjie Wang, Zhimei Sun

**Affiliations:** 1 School of Materials Science and Engineering, Beihang University, Beijing 100191, China; ydhuang@buaa.edu.cn (Y.H.); jzhou@buaa.edu.cn (J.Z.); gjwang@buaa.edu.cn (G.W.); 2 Centre for Integrated Computational Materials Engineering, International Research Institute for Multidisciplinary Science, Beihang University, Beijing 100191, China

**Keywords:** phonon, temperature-dependent behavior, vibration density of states, anharmonic interactions

## Abstract

The vibrational density of states (VDOS) of solids in the low-energy regime controls the thermal and transport properties of materials, such as heat capacity, heat conduction, free energy and entropy. In α-Cristobalite, the low-frequency part of vibration density of states (VDOS) has many common features with the Boson peak in silica glass of matched densities. Recent theoretical work reported that anharmonic phonon–phonon interactions were critical for the low-frequency part of VDOS in α-Cristobalite. Therefore, it is urgent to identify the role of different anharmonic interactions from first principles. In this paper, we focus on the main peak of the low-frequency part of VDOS in α-Cristobalite. Calculated by our own developed codes and first principles, we find that the quartic anharmonic interaction can increase the frequency of the peak, while the cubic anharmonic can reduce the frequency and change the shape of the peak. Meanwhile, the anharmonic interactions are critical for the temperature effect. Therefore, we calculated the temperature-dependent property of the peak. We find that the frequency of the peak is directly proportional to the temperature. The atomic displacement patterns of different temperatures also confirm the above conclusion. All our calculations converged well. Moreover, our basic results agree well with other published results. Finally, we highlight that our codes offer a general and reliable way to calculate the VDOS with temperature.

## 1. Introduction

The vibrational spectra of solids play important role in thermodynamics physics [1,2], where the low-energy vibrational spectra are described by the Debye model as proportional to phonon frequency squared [3,4]. Boson peak is an anomaly in VDOS (vibration density of states) that appears in glasses upon normalizing the VDOS g(ω) by the Debye law ω^2^ [5]. Therefore, Boson peak is the characteristic anomalous behavior of the low-frequency part of VDOS [6]. In early years, there was consensus that Boson peak, which is usually observed in disordered crystals and amorphous phases, was the feature of disorder [7,8]. Hence, based on disorder, many theories were developed to explain the origins of Boson peak in glasses [9,10,11,12,13,14,15,16,17,18]. In 2014, in some ordered crystals, for example, α-Cristobalite, α-Quartz, Coesite, the low-frequency part of VDOS had many common features with Boson peak in silica glass [19,20,21]. According to the published experiments, for α-Cristobalite and silica glass with matched densities, the DOS of silica glass appeared as the smoothed counterpart of DOS of the corresponding crystal, which illustrates that two systems have the same number of the excess states relative to the Debye model, the same number of states in the low-energy region, and the same specific heat. In 2015, the similarity was attributed to the atom displacement patterns of α-cristobalite. A recent theoretical paper reported that anharmonic phonon–phonon interactions are critical for the low-frequency part of the VDOS in α-cristobalite [22]. Therefore, it is urgent to identify the role of anharmonic interactions for the low-frequency part of VDOS in α-cristobalite from first principles.

Unraveling the anharmonic interactions of VDOS in α-Cristobalite remains as a challenge. Recently, the phonon Green function method by using elastic constants was used to calculate the VDOS at the low-frequency region [22]. However, we found that the method completely fails in α-Cristobalite. The ratio of shear modulus to bulk modulus G/K is 2.38 and the Poisson ratio lies in the vicinity of the value −0.2, which led to wrong phonon velocity and frequency [22,23,24,25,26,27]. Therefore, we developed the codes for phonon Green functions from first principles to calculate the VDOS accurately. Because the anharmonic interactions include the phonon–phonon cubic and quartic interactions, we must identify the role of different phonon–phonon interactions for the VDOS. In detail, we focus on the main peak of the low-frequency part of VDOS in α-Cristobalite. We present that the quartic interaction can induce frequency shift of the peak, while the cubic interaction can simultaneously change both the frequency and shape of the peak. However, the effect of the cubic interaction is weak. In fact, the anharmonic interactions are critical for temperature effect. Therefore, we show the temperature-dependent behavior of the peak. The frequency of the peak shifts to higher frequency and is directly proportional to temperature. Moreover, we also show the Transverse Acoustic (TA) branches’ dispersion for different temperatures. Meanwhile, we present that the atom displacement patterns of α-Cristobalite are similar at different temperatures. We find that the atoms have similar vibration direction, but different vibration amplitude. This result agrees well with the temperature-dependent behavior of the VDOS. In conclusion, we identify the roles of anharmonic interactions for the VDOS and offer a way to calculate VDOS accurately.

## 2. Methods

Generally, the phonon–phonon interactions include the following terms:

(1) U=harmonic+cubic+quartic+⋯

where the second and third terms are anharmonic interactions and the last plus (+) means even higher orders that are normally neglected. We use 4 × 4 × 4 supercells and Perdew−Burke−Ernzerh of exchange-correlation functional with 38Ry cut-off to calculate the phonon dispersions with harmonic approximation in α-Cristobalite by using Alamode-1.1.0 [28,29,30] and Quantum Espresso-6.4.1 [31,32]. And we use 2 × 2 × 2 supercells with 70Ry cut-off to calculate the cubic and quartic anharmonic interaction force constants (IFCs). The displacement distance of atoms is chosen as 0.03 Å. In the self-consistent phonon (SCPH) calculation of α-Cristobalite, we use the parameters 2 × 2 × 2 of kmesh_scph and kmesh_interpolate to generate the effective second order IFCs with different temperatures. In addition, the off-diagonal elements of the loop diagram are neglected in the SCPH calculation. The vibration density of states is calculated with 30 × 30 × 30 q-points. However, there are no available codes to calculate VDOS, including all the various phonon–phonon interactions. Here, for the first time, we developed some codes to achieve this goal. The following are algorithms for the calculations. Firstly, from the calculated phonon–phonon interactions we can obtain the phonon eigenfrequency by [33,34]:

(2) $$\Omega_{\lambda }=\omega_{\lambda }^{\left(2\right)}+\Delta \omega_{\lambda }^{\left(3\right)}+\Delta \omega_{\lambda }^{\left(T\right)}+\Delta \omega_{\lambda }^{\left(4\right)}$$

Here λ=(q,j), *q* is the q-point index and *j* is the phonon band index. $\omega_{\lambda }^{\left(2\right)}$ is the phonon frequency in harmonic approximation. $\Delta \omega_{\lambda }^{\left(3\right)}$. and $\Delta \omega_{\lambda }^{\left(T\right)}$ are frequency corrections given by the cubic anharmonic, and $\Delta \omega_{\lambda }^{\left(4\right)}$ is a frequency correction provided by the quartic anharmonic. The phonon linewidth $\Gamma_{qj}$ can be deduced from the cubic anharmonic interaction [35,36]. Now considering the anharmonic interactions, we can construct the phonon propagators by the Green functions [22,37]:

(3) $$G_{qj}\left(\omega \right)=\frac{1}{\omega^{2}-\Omega_{qj}^{2}+i\omega \Gamma_{qj}}$$

Here the eigenfrequency $\Omega_{qj}$ that includes the cubic and quartic anharmonic and the phonon linewidth $\Gamma_{qj}$ are used to construct the phonon propagators. Finally, the VDOS is calculated by the imaginary part of the propagators:

(4) $$g\left(\omega \right)=\frac{-2\omega }{3N}\underset{qj}{\sum }ImG_{qj}\left(\omega \right)=\underset{qj}{\sum }\frac{-2\omega }{3\pi N}\times \frac{-\omega \Gamma_{qj}}{{\left(\omega^{2}-\Omega_{qj}^{2}\right)}^{2}+{\left(\omega \Gamma_{qj}\right)}^{2}}$$

The summation includes all the q-points and phonon branches. N is the number of atoms in the unit cell. The weights for different q-points have been considered. The phonon Green functions codes for calculating VDOS will be released later. The calculation equipment is the computer cluster, which is composed of Intel(R) Xeon(R) Silver 4110 CPU.

## 3. Discussion and Results

**Figure 1:**
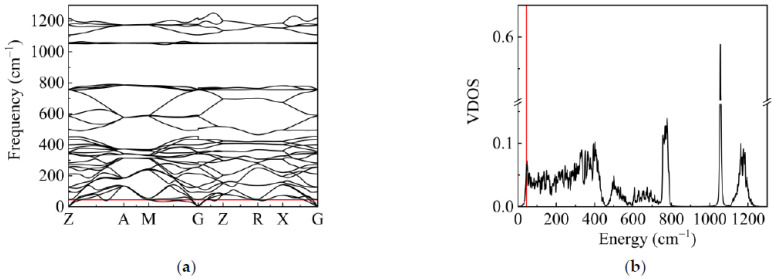
(**a**) The Phonon dispersion of α-Cristobalite. The red line is the frequency of the main peak at 44 cm^−1^. (**b**) The vibrational density of states (VDOS) of α-Cristobalite with harmonic approximation. The red line is the location of the main peak.

We now applied the above algorithms and developed codes to calculate VDOS of α-Cristobalite. Firstly, only harmonic approximation was considered to calculate the phonon dispersion and VDOS. Our calculation results with harmonic approximation agree well with the reference [38]. The system is stable under harmonic approximation (Figure 1a), and the VDOS is shown in Figure 1b. We focused on the main peak of the VDOS at the low-frequency part. The peak of VDOS in crystal theoretically is an acoustic Van Hove singularity. The Van Hove singularity is the shoulder of the phonon dispersion where the density of states is not differentiable. Hence, from Figure 1a,b, it is observed that the frequency of the peak is indeed TA singularity (Van Hove singularity) at M (0.5,0.5,0). The Van Hove singularity usually results from the piling up of the vibrational states near the boundary of the Brillouin zone. The M point is indeed at the boundary of the Brillouin zone. Therefore, the vibration states at M (0.5,0.5,0) are the main contribution to the peak. We will focus on the vibration states at M (0.5,0.5,0) to check the roles of the anharmonic interactions.

**Figure 2:**
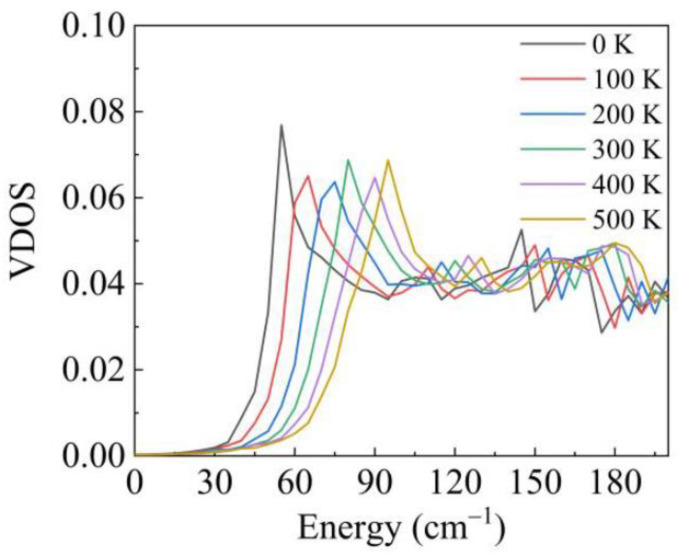
Considering quartic anharmonic, VDOS of α-Cristobalite with different temperatures at the low-frequency region.

**Figure 3:**
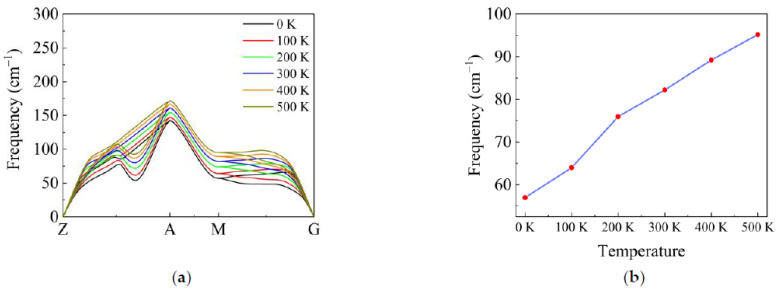
(**a**) The dispersion of TA branches with increasing temperature. (**b**) The frequency of the main peak of the VDOS with increasing temperature. Every point corresponds to the frequency of TA branches’ shoulder at M (0.5,0.5,0).

**Figure 4:**
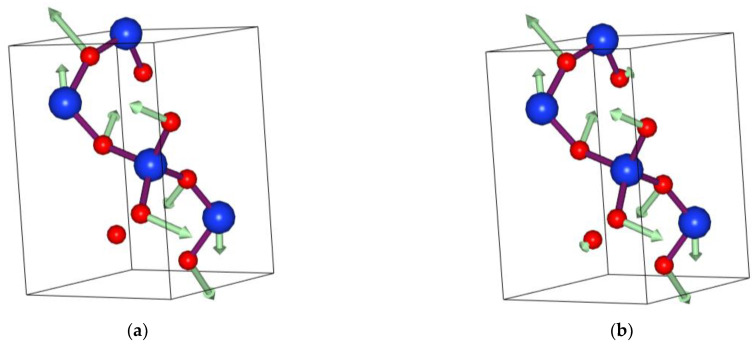
(**a**) Atom displacement patterns at 0 K. (**b**) Atom displacement patterns at 300 K. The vibration directions are depicted by green arrows, on an arbitrary scale for better visualization. The red balls are O atoms. The blue balls are Si atoms.

The anharmonic interactions generally include the phonon–phonon cubic and quartic interactions. Firstly, we consider the quartic interaction to calculate the VDOS at different temperatures. Later, we will check the role of the cubic interaction and show that the effect of the cubic interaction on the VDOS is weak. We used the SCPH algorithm to generate the effective harmonic force constants for considering the quartic interaction. The low-frequency part of the VDOS at different temperatures has been plotted in Figure 2. Because the phase change happens in α-Cristobalite at around 520 K, the maximum temperature was limited to 500 K. It is observed that the main peak shifts to higher frequency with increasing temperature in Figure 2. We show the TA branches’ dispersion and frequency of the peak with increasing temperature in Figure 3. It is obviously seen that in Figure 3a the shoulder of the TA branches at M (0.5.0.5 0) shifts to higher frequency, which corresponds to the shift of the main peak. Figure 3b illustrates that the frequency of the main peak increases in direct proportion to temperature. Meanwhile, we present the atom displacement patterns at M (0.5,0.5,0) with 0 K and 300 K in Figure 4. The atom displacement pattern of 0 K agrees well with the published result [39]. It is clear to see that the vibration direction of the atoms changes little in Figure 4. This phenomenon can be explained from two perspectives. According to the lattice dynamic, the amplitude of the vibration is related to the frequency of phonon. Therefore, it is reasonable that the atoms should have larger vibration amplitude with increasing temperature. Moreover, the SCPH algorithm considers first order contribution to the phonon self-energy from the quartic interaction. According to the perturbation theory analysis, the first order phonon self-energy from the quartic interaction is the real number, which mainly contributes to the frequency correction. Thus, the frequency of phonon increases with increasing temperature. Meanwhile, the eigenvector of phonon that is related to the vibration direction of atoms changes little.

**Figure 5:**
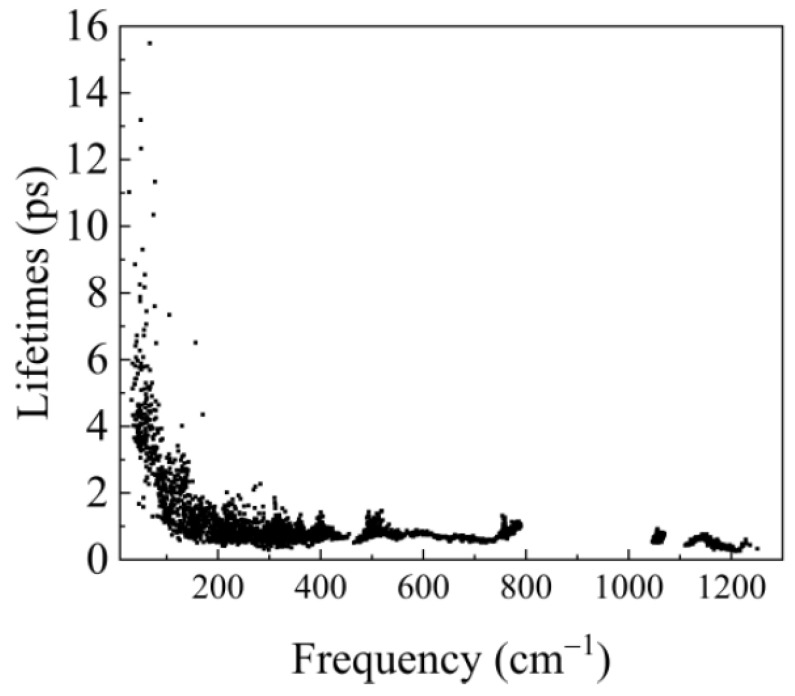
The phonon lifetimes due to the phonon–phonon cubic interaction at 300 K.

Another physical quantity of interest is the cubic anharmonic interaction [40]. The cubic interaction can give both frequency shift and phonon lifetimes as the forms of bubble and tadpole. Here, we consider both tadpole and bubble contributions to phonon frequency based on the perturbation theory [41,42]. The frequency shift of tadpole and bubble is given by [33],

(5) $$\Delta \omega_{\lambda }^{\left(T\right)}=\frac{\hbar }{8\omega_{\lambda }}\underset{\lambda_{1}}{\sum }\underset{\nu_{2}}{\sum }V_{\lambda ,-\lambda \left(0,\nu_{2}\right)}^{\left(3\right)}V_{\lambda_{1},-\lambda_{1},\left(0,\nu_{2}\right)}^{\left(3\right)}\times \frac{2n_{1}+1}{\omega_{1}\omega_{2}{\left(\omega_{2}\right)}_{P}}$$

(6) $$\begin{array}{c} \Delta \omega_{\lambda }^{\left(3\right)}=\frac{\hbar }{16\omega_{\lambda }}\underset{\lambda_{1}\lambda_{2}}{\sum }{\left|V_{\lambda -\lambda_{1}-\lambda_{2}}^{\left(3\right)}\right|}^{2}\delta_{q-q_{1}-q_{2}}\times \frac{n_{1}+n_{2}+1}{\omega_{1}\omega_{2}{\left(\omega_{\lambda }-\omega_{1}-\omega_{2}\right)}_{P}}\\ \;\;\;\;\;\;\;\;\;\;\;\;\;\;\;\;\;\;\;\;\;\;\;+\frac{\hbar }{16\omega_{\lambda }}\underset{\lambda_{1}\lambda_{2}}{\sum }2\left|V_{\lambda \lambda_{1}-\lambda_{2}}^{\left(3\right)}\right|\delta_{q+q_{1}-q_{2}}\times \frac{n_{1}-n_{2}}{\omega_{1}\omega_{2}{\left(\omega_{\lambda }+\omega_{1}-\omega_{2}\right)}_{P}} \end{array}$$

where, *n* is the Boson–Einstein distribution and *V^(3)^* is the potential obtained by the cubic anharmonic IFCs [43]. δ is the Dirac function. ω is the phonon frequency. The subscript λ=(q,v), q refers to the phonon wave vector and v represents phonon dispersion branch. ℏ is the reduced Planck constant. The Cauchy principle value $\frac{1}{{\left(x\right)}_{p}}=\frac{x}{x\left(x^{2}+\varepsilon^{2}\right)}$, where the infinitesimal *ε* is a broadening factor. Meanwhile, the cubic anharmonic can provide phonon linewidth $\Gamma_{\lambda }^{\left(3\right)}\left(\omega \right)$. We present the phonon–phonon scattering results from the cubic anharmonic interaction in Figure 5. The result agrees well with the published paper [38].

**Figure 6:**
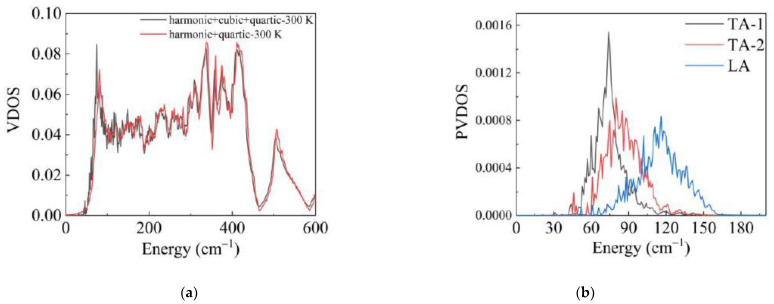
(**a**) Red line is the VDOS of α-Cristobalite with the quartic anharmonic interaction. The black line is the VDOS with the cubic and quartic anharmonic interactions. (**b**) Partial vibration density of states (PVDOS) of α-Cristobalite with the cubic and quartic anharmonic interactions.

Then, we used the phonon propagator algorithm to calculate the VDOS with the phonon lifetimes and frequency correction. Although the cubic interaction contributes little to the main peak of the low frequency part, the effects of the cubic interaction are important. The cubic anharmonic interaction can lead the frequency of the peak shifts to lower frequency as shown in Figure 6a. In addition, it is noticed that the shift of frequency mainly appears at the low-frequency part. Due to the limited lifetimes, there is a slight change on the shape of the low-frequency part of the VDOS. Furthermore, if the cubic anharmonic interaction is strong, i.e., phonon–phonon scattering is strong [43,44,45], the frequency of the peak may be overestimated without the cubic anharmonic interaction. These calculation results identify the role of the cubic anharmonic interaction for the VDOS. It is seen in Figure 6b that the peak is predominantly contributed from TA phonon branches, while the Longitude Acoustic (LA) branch contributes a little to the peak. This result agrees well with the analysis of the harmonic phonon. Moreover, we checked the error of the calculation data. The experiment at room temperature shows that the frequency of the peak is about 7.06 meV [39]. However, our calculation result at 300 K is about 8.92 meV. The lattice parameters in our calculation are 5.103 and 7.135 angstrom, which are different from the experiment value 4.978 and 6.948 angstrom [39]. Due to the different lattice parameters, the error can be understood.

Finally, we discuss the calculation method. As usual, for the fast calculation, the elastic constants can be used to generate the sound velocity [22,46]:

(7) $$c_{T}^{2}=\frac{\mu }{\rho },c_{L}^{2}=\frac{K+\frac{2\left(d-1\right)}{d}\mu }{\rho }$$

*μ* is the shear modulus, *K* is the bulk modulus, *d* is the dimension, *ρ* is the density. The sound velocity will be used to calculate the phonon frequency:

(8) $$\omega_{L,T}=c_{L,T}q-iD_{L,T}q^{2}$$

where *q* is the wave vector, *D_L,T_* is the constant. Combing the frequency and *D_L,T_*, the phonon propagators can be constructed to calculate the low-frequency region of the VDOS. However, this method does not work well in α-Cristobalite. According to the experiment and calculation from first principles, the ratio of the shear modulus to the bulk modulus μ/K is 2.38. Moreover, the Poisson ratio lies in the vicinity of the value −0.2. Furthermore, using the Equation (7), the phonon velocity of the TA is about 417.26 m/s. However, the average phonon velocity of the TA along the symmetry path is 2402.08 m/s. Hence, it is evident that the calculation method of the VDOS by using shear and bulk modulus is not reliable.

## 4. Conclusions

In conclusion, we studied the lattice dynamics of VDOS with increasing temperature in α-Cristobalite. We developed the codes to use the phonon Green function method to calculate the VDOS. This way can include different phonon–phonon interactions. We calculated the low-frequency part of the VDOS with increasing temperature, which shows that the frequency of the main peak at low-frequency part increases in direct proportion to temperature. This temperature-dependent behavior can be explained from phonon-self energy analysis and atom displacement patterns. Furthermore, we identified the roles of cubic and quartic interactions for the main peak at the low-frequency part of VDOS in α-Cristobalite. We report that the quartic anharmonic can increase the frequency of the peak, while the cubic anharmonic can reduce the frequency of the peak and change the shape of the peak. Furthermore, the effect of the cubic interaction is weak in α-Cristobalite. Moreover, we confirmed that the calculation method by using the elastic constants may obtain the unreasonable phonon velocity which leads to the unreasonable VDOS. Over all, our work provides the temperature-dependent behavior of VDOS in α-cristobalite from first principles and identifies the role of the cubic and quartic interactions for the VDOS. Our codes provide a general and reliable calculation method for VDOS, including different anharmonic interactions.

## Data Availability

The data presented in this study are available on request from the corresponding author. The data are not publicly available due to the huge amount of data.
